# Pathological Chemistry—Researches into the Elimination of the Bromides, &c.

**Published:** 1869-01-01

**Authors:** 


### CHICAGO MEDICAL JOURNAL.

*Vol. XXVI.—JANUARY* 1, 1869.—*No.* 1.

### PATHOLOGICAL CHEMISTRY.

### *Researches into the Elimination of Bromides, and into the presence of Bromine, normally in the organism.*

REPORTED TO THE BIOLOGICAL SOCIETY (PARIS), BY DR. RABUTEAU.

ALTHOUGH I have already published, in the "Gazette Hebdomadaire," of the 24th of April, the process which I employ in order to detect traces of a bromide in the urine, I think I may repeat it here. I add a small quantity of pure soda to the urine, then I evaporate it to dryness. The residue is next heated to redness, in a porcelain capsule, then treated with distilled water. After filtration, I obtain a liquor as clear as spring-water, in which it is easy for me to detect the bromides which it may contain, by pouring upon it *Nitric acid*, which liberates the bromine, and by collecting this last by the aid of sulphuret of carbon. In proportion as the bromide exists in greater or less quantity, the sulphuret of carbon is colored an intense red, or an orange yellow.

This process is long and extremely difficult, but it is consistent with the greatest delicacy. In fact, if 500 grammes of urine be evaporated, and if the liquor resulting from the washing of the residuum amount to only ten cubic centimetres, it is possible to detect even the $\frac{1}{1500000}$ part of bromine.

I published, likewise, the principal results of my investigations into the metamorphoses, and the mode of elimination of bromates, and it will be perceived that I have detected bromides in my own urine, and in that of dogs and of rabbits, several days after the absorption of small quantities of bromates. These facts have constituted for me the point of departure for new investigations into the elimination of bromides.

On the 3rd of March, I took, fasting, one gramme of *Bromide of Potassium*, dissolved in fifty grammes of water. From the tenth minute I found bromine in my urine, and my saliva; but what astonished me was, that on the 20th of March I obtained a beautiful orange yellow color, with sulphuret of carbon, after having evaporated 100 to 150 grammes of urine. Subsequently to this date, I was able to evaporate larger quantities, 300 grammes for example, but then bromine was constantly detected, even at the end of fifty-two days. On the other hand, the urine of a dog, which I had cured of saturnine intoxication, by the aid of *Bromide of Potassium*, manifested the presence of bromine after two months, so that I regretted infinitely not having analyzed my urine, and that of my dog, before taking the medicine. I then examined the urine of a great number of persons; I even brought specimens of urine from the country, *and in all I found bromine, when I had evaporated from three to four hundred grammes of it.* I could not detect any of it, when I experimented upon only one hundred to one hundred and fifty grammes. These different experiments repeated nearly two hundred times, have induced me *to conclude that bromine exists normally in the organism;* as to that which has been found by evaporating only one hundred to one hundred and fifty grammes of urine, it may be said that it originates from the administration of a bromide, and as I have found it under these circumstances in my own case, during three weeks, in the case of a rabbit during sixteen days, in that of another rabbit during twenty-five days, after the ingestion of *Bromide of Potassium*, or of different bromates, I may draw this con clusion that *the bromides are eliminated slowly*.

I wrote, on the 24th of April, in the "Gazette Hebdomadaire," that the bromide of potassium appeared during more than a month in the urine, and in the saliva, even when the dose absorbed had been only one gramme. I did not imagine then that bromine existed normally in the organism; I believe to-day, that one gramme of bromide of potassium would disappear at the end of three weeks, and I base my belief upon this, that, under ordinary circumstances, bromine is not found in the urine, when only one hundred and fifty grammes of this liquid have been evaporated, at least when only a certain dose of it has been absorbed with any special intention. As to the bromine which is detected always after having evaporated three to four hundred grammes of urine, I call it normal bromine, and it penetrates every day into the organism by alimentation.

What is the origin of this normal? I am actually engaged in investigations into this subject, and I propose to report to the Biological Society the results at which I shall arrive.

M. Laborde insisted upon the difficulty of the investigation of bromine in the urine, the coloring matters of this liquid being capable of inducing error.

M. Rabuteau said that, in fact, the examination was very difficult, even when bromine has been artificially added to the urine. It is for this reason that he would proceed by incineration preferably.

M. Chalvet would desire that for this demonstration of bromine in the normal state in the urine, we should not be contented with reactions; he should desire that the metalloid be isolated.

M. Rabuteau subsequently communicated the results which he had obtained from the ingestion of *Perchlorate of Potassium*, the most noticeable of which was retardation of the pulse.

### PHYSIOLOGICAL AND PATHOLOGICAL CHEMISTRY.

I. *Sulphate of Quinine* does not diminish the urea.

II. Physiological studies of *Perchloride of Potassium.*

The use of the salt against intermittent fevers,—by Dr. Rabuteau.

It is well known that *Arsenic* is employed successfully against simple intermittent fevers, and that it diminishes the urea to a considerable extent. I could cite upon this subject different experiments made upon well fed dogs, in which I have seen this element diminish from 65 to 16 parts per 1000, under the influence of a few centigrammes of *Arsenite of Potassium,* then increase to the normal amount after the elimination of the poison.

Is this the case, likewise, with *Sulphate of Quinine?* It seems not, from some experiments already made, and in order to contribute to the elucidation of the question, I have made the following investigations, at the suggestion of one of my preceptors, M. Sée, Professor of the Faculty of Médicine.

*First Experiment.*—1*st. Sulphate of Quinine.*—On the 9th of May, at 9.30 P. M.. I took one gramme of *Sulphate of Quinine.* At 11 o'clock I commenced to experience ringing in the ears; my intellect became less clear; I went to bed, and slept a little, with the design to experience more fully the effects of the medicine. When I arose, I observed a real titubation. The pulse was retarded, but what attracted my attention much more than the retardation, was the weakness of the cardiac impulse. The next day, at 6 o'clock, the tinnitus aurium still persisted, it diminished gradually, and at 8 o'clock was no longer perceptible. If I enter into the detail of well known facts, it is because I have experienced the same phenomena, except the tinnitus aurium, after having taken five grammes of *Perchloride of Potassium.* I shall soon have to speak of this new drug, destined, perhaps, to future importance. I had measured the urea eliminated during the two preceding days; I collected my urine at different intervals, and analysis demonstrated that the *Sulphate of Quinine* had occasioned no variation in the quantity of urea, which the following figures demonstrate:

| DAYS. | | Urine of 24 hours. | Urea per 1000. | Total urea eliminated during 24 hours. |
|---|---|---|---|---|
| May 7th, up to 6 P. M., | | 10.85 | 20.12 | 21 83 |
| May 8th to 9th, | | 9.65 | 22.65 | 21.85 |
| | Urea per cent. | | | |
| From May 9th to May 10th. | From 6 to 9 P. M. | 16.76 | | |
| | " 9¾ to 10½, | 25.88 | | |
| | " 10½ P. M. to (May 10th) 5 A. M. | 28.82 | | |
| | " 5 to 8½ A. M. | 20.88 | | |
| | " 8½ to 12 M. | 20.88 | | |
| | " 12 to 6 P. M. | 19.70 | | |
| (From May 9th to May 10th, total) | | 10.71 | 22.06 | 23.62 |
| From 10th to 11th. | May 10th, from 6 to 9 P. M. | 18.23 | | |
| | From 9 P. M. to 9 A. M., May 11th, | 25.87 | | |
| | From 8 A. M. to 6 P. M. | 20.88 | | |
| (From 10th to 11th, total) | | 9.70 | 23.53 | 22.82 |
| From May 11th to 12th, | | 10.80 | 23.50 | 25.38 |
| " " 12th to 13th, | | 11.60 | 18.23 | 21.15 |
| " " 13th to 14th, | | 11.20 | 20.00 | 22.40 |

The figures of the third column present considerable differences, which are very easily explained, for it is known that urea exists normally in larger quantity in the urine of the night.

*Second and Third Experiments.*—These were made upon a dog of large size, whom I had caused, during the early portion of March, to swallow ten grammes of *Bromide of Potassium*, with a double intention. In the first place, I desired to cure him of acute saturnine intoxication, caused by twenty centigrammes of *Acetate of Lead*, and in the second place, to study the elimination of the bromide. This dog had been immediately cured, and his health had remained perfect; I had, moreover, nourished him perfectly.

From the date of the 8th of May, I gave him four hundred grammes of cooked meat, and two hundred grammes of bread, daily, at 6 o'clock, P. M. On the 10th of May, at 4 o'clock, P. M., I made him swallow two grammes of *Sulphate of Quinine*, mixed with a little food, but he left a portion of the food; at this time I observed already a certain dullness, which became more and more marked. At 7 o'clock the cardiac pulsations became less numerous; the dog walked unsteadily; he gazed at me in a most singular fashion. At 9 o'clock he vomited the greater portion of the food which he had swallowed. I noted this fact, for it explained the diminution of the urea found the next day. The succeeding days his health was perfect.

On the 17th and 18th of May, at 2 o'clock, P. M., I made him swallow one gramme of *Sulphate of Quinine*, thinking that in this dose that salt would not cause vomiting. My conjectures were verified.

Below are the results which have been furnished me by analyses of the specimens of urine collected at different periods:

| Days. | Urea per 1000. |
|---|---|
| May 9th, 9 A. M., | 51.47 |
| " 10th, " | 50.69 |
| " 10th, 4 P. M., before the experiment, | 47.05 |
| " 11th, 9 A. M., | 36.18 |
| " 12th, " | 52.94 |
| " 13th, " | 55.80 |
| " 14th, " | 58.21 |
| " 15th, " | 57.35 |
| " 16th, " | 60.00 |
| " 17th, " | 60.00 |
| " 17th, 6.45 P. M., | 57.35 |
| " 18th, 9 A. M., | 63.82 |
| " 18th, 7 P. M., | 53.82 |
| " 19th, 9 A. M., | 57.35 |
| " 20th, " | 58.25 |
| " 21st, " | 57.90 |
| " 22nd, " | 50.00 |
| " 23rd, " | 60.00 |
| " 24th, " | 58.82 |

The preceeding figures exhibit some marked variations, but they prove no diminution of the urea under the influence of the *Sulphate of Quinine*, for the differences observed are readily explained. The lowest figure is 36.18, but the dog had vomited his food the preceding evening, and had eaten nothing since. Other sensible differences appear between the figures furnished by the analysis of the specimens of urine of the morning and the evening, now it is known that the former contains normally a greater quantity of urea. This is what I have frequently observed. Since then the urea is not diminished, since on the other hand the *Sulphate of Quinine* is antipyretic, reduces the temperature, and consequently diminishes combustion, it is absolutely necessary to seek elsewhere for the deficit in the products of this very combustion. It is extremely probable that this deficit bears upon the carbonic acid; but I have not yet investigated this subject.

2. *Perchlorate of Potassium* ($Kclo^4$).—This salt, when it is perfectly pure, presents itself under the appearance of a white powder, composed of small prismatic crystals. It is about three times less soluble than the *Chlortae of Potassium* (?), but it is much more permanent than this last, for it needs a much higher temperature to effect its decomposition into *Chlorate of Potassium* (?), and into oxygen.

The *Perchlorate of Potassium* of commerce is not pure; the specimens which I have had under examination have contained nearly two-thirds of their weight of chlorate. In order to purify it, I at first proposed to treat it with *Sulphuric acid*, which has the effect to destroy the chlorates. This process is a good one, but only when there was only a small quantity of the chlorate. I then devised another process, based upon the property which *Hydrochloric acid* possesses of decomposing chlorates, by producing an evolution of chloro-chloric (?) gas, and leaving the chlorides as residue.

I treated the impure perchlorates with boiling *Hydrochloric acid*, diluted with half its weight of water. There is disengaged, when cooled, a yellow vapor of a peculiar odor, recalling that of chloric acid, or of the peroxyd of chlorine. The liquid is tested from time to time, and when it no longer discolorizes indigo, from the effect of the *Sulphurous acid*, it is certain that the perchlorate is freed entirely from the chlorate. It only remains to separate the perchlorate by decantation, and to treat the residuum with boiling distilled water, which dissolves the chloride and leaves the perchlorate, which is but slightly soluble. This process necessitates the loss of a certain quantity of the material, but the residuum obtained is perfectly pure.

But how shall we detect the presence of the perchlorates in the urine? I have sought in vain thus far a simple and accurate process. I have hence been compelled to adopt the following, which presented itself naturally to my mind. I precipitate, first, the natural chlorides, by means of *Nitrate of Silver*, I then boil the liquid and filter it. I remove next, the excess of *Nitrate of Silver*, then boil anew and filter a second time. The clear liquid thus obtained is evaporated to dryness; the residuum is next heated to redness, in order to transform the perchlorate into chloride, which I effect easily, by the ordinary methods. The process is long but accurate.

My investigations into the *Perchlorate of Potassium* consists of preliminary experiments made upon animals, and of experiments made upon man, with the view to study the physiological effects of this salt, and its mode of elimination. I will say nothing of the first investigations, which will be published hereafter, but will pass immediately to the second series of experiments.

1st. On the 3rd of July, at 4 P. M., I took five grains (grains or grammes?) of *Perchlorate of Potassium*, partly dissolved, partly suspended, in fifty grains (?) of water, then I drank fifty grammes of pure water. The taste of the salt was exceedingly feeble, and, although the undissolved portion was necessarily brought into direct contact with the gastric surface, I experienced no epigastric sensation. But I was soon strangely surprised; I experienced sensations similar to those produced by *Sulphate of Quinine*, except the tinnitus aurium. My gait was staggering; I felt a heaviness of the head, especially in the frontal region; my ideas were confused; the pulse was retarded, and the natural heat appeared to be diminished. Toward half past seven this intoxication, which I termed chloric intoxication, diminished perceptibly, and at 8 o'clock it had disappeared.

My urine and saliva were collected at tolerably frequent intervals, in order to be subjected to analysis. I perceived, also, that the salt was rapidly eliminated, that it appeared after ten minutes in these two liquids, and that it could no longer be detected after forty-eight hours. I determined, moreover, that it produced some diuretic effect, and that it did not diminish the quantity of urea; this last property establishes one more analogy between *Perchlorate of Potassium* and *Sulphate of Quinine.* The results of my analysis are comprised in the following tables:

| DAYS. | Urine of 24 hours. | Urea per 1000. | Total urea eliminated in 24 hours. |
|---|---|---|---|
| From July 1st to 2nd,.......... | 9.00 | 21.20 | 19.08 |
| " " 2nd to 3rd,.......... | 9.60 | 18.23 | 17.50 |
| " " 3rd to 4th,.......... | 12.26 | 16.40 | 20.10 |
| " " 4th to 5th,.......... | 9.20 | 23.70 | 21.83 |
| " " 5th to 6th,.......... | 9.15 | 22.17 | 20.28 |
| " ' 6th to 7th,.......... | 10.25 | 20.15 | 20.65 |

*Perchlorate of Potassium against Intermittent Fever.*—The curious effects produced by this salt, this intoxication, comparable to that induced by *Sulphate of Quinine*, have suggested to me the idea that the *Perchlorate of Potassium* might be useful in intermittent fevers. I also desired earnestly to embrace the opportunity to employ it, whenever it might present itself to me.

*Second Experiment.*—A young man, twenty-five years of age, had two years previously contracted intermittent fever in Wallachia. Since his return to France he had been subject, about every six months, to daily recurrences, which he controlled by *Sulphate of Quinine.*

On the 21st of July a paroxysm recurred; he consulted me at that time, and informed me that he would have a similar paroxysm on the following day about 3 P. M., unless he should take *Sulphate of Quinine.* As yet he had taken no medicine. I gave him five grammes of *Perchlorate of Potassium*, which I had myself purified, and directed him to take this dose, with water, at once, the next day at 2 o'clock, which was done. *The fever did not return;* but there were substituted for it symptoms similar to those which I had already experienced. My patient perceived his gait become unsteady; he was obliged to grasp the railing of a staircase in order to descend it; his ideas became confused, and his head was dull. All disappeared toward 6 o'clock, that is to say, four hours after the ingestion of the drug. Every thing had occurred as in my own case. It is extremely probable that if I had examined my patient during this time, I would have found his pulse retarded.

If the febrifuge properties of *Perchloride of Potassium* should be verified, therapeutics would possess a new and valuable agent, especially when it might be necessary to act promptly. We know that the effects of *Sulphate of Quinine* are not developed immediately, and that we find ourselves helpless in the presence of an attack of pernicious fever, as the *Perchlorate of Potassium* acts almost as soon as it is absorbed, it merits special investigation. I insist upon but one single condition, *upon the use of a pure article;* for the perchlorate of commerce contains a large proportion of chlorate. I have designated the mode of separating it from this latter salt.

M. Láborde was ignorant whether the therapeutic action o f *Perchlorate of Potassium* had been previously studied, it is known from the investigations of M. Socquet (de Lyon) that it was hyposthenetic.

M. Isambert also believed that the *Perchlorate of Potassium* had never been the subject of physiological or clinical investigation, as to the researches of M. Socquet upon *Chlorate of Potassium*, they do not appear to demonstrate very clearly the hyposthenetic action of this salt.

